# Supplementary material for: Compound-SNE: comparative alignment of t-SNEs for multiple single-cell omics data visualization
Source: Bioinformatics. 2024 Jul 25;40(7):btae471. doi: 10.1093/bioinformatics/btae471 (PMC11290359; doi:10.1093/bioinformatics/btae471)
Supplement: btae471_Supplementary_Data [file btae471_supplementary_data.pdf]

SUPPLEMENTARY INFORMATION:

Compound-SNE: Comparative alignment of t-SNEs for multiple single-cell omics data visualisation

Colin G. Cess[1] and Laleh Haghverdi[1]

1 Max-Delbrück-Center for Molecular Medicine in the Helmholtz Association (MDC), Berlin Institute for Medical Systems Biology (BIMSB), Berlin, Germany

1 Method details

1.1 Implementation of alignment forces

As described in the main text (and rewritten here), Compound-SNE functions to minimize both normal t-SNE loss (calculated as the KL divergence of the probability distributions in high dimensional space and embedding space) and the distance between cell type centers in embedding space.

$$d_i = (Y_{r,center_i} - Y_{s,center_i})^2$$

$$L_{total} = L_{tsne} + \sum_{i=1}^{K} \lambda_i d_i$$

where d_i is the squared distance between embedded reference centers (of K clusters), $Y_{r,center_i}$, and embedded sample centers, $Y_{s,center_i}$, for the cell type i. Minimizing this Loss function with respect to the position y_j of each cell j in the embedding space, requires calculation of the derivative:

$$\frac{\mathrm{d}L_{total}}{\mathrm{d}y_j} = \frac{\partial L_{total}}{\partial y_j} + \frac{\partial L_{total}}{\partial d_i}\frac{\partial d_i}{\partial y_j} = \frac{\partial L_{tsne}}{\partial y_j} + 2\lambda_i (Y_{r,center_i} - Y_{s,center_i}) \frac{\partial Y_{s,center_i}}{\partial y_j}$$

Because $Y_{s,center_i} = \frac{1}{N_i}\sum_{i=j}^{N_i} y_j$ if cell j belongs to cluster i (with N_i the number of cells in that cluster), it follows:

$$\frac{\mathrm{d}L_{total}}{\mathrm{d}y_j} = \frac{\partial L_{tsne}}{\partial y_j} + 2\frac{\lambda_i}{N_i}(Y_{r,center_i} - Y_{s,center_i})$$

The first term just presents the standard $t-$SNE derivative, according to which its gradients descend forces act. In the standard $t-$SNE the location of the data point (cell j) in embedding space are updated in iteration t by:

$$y_j^{(t)} = y_j^{(t-1)} + \eta \frac{\partial L_{tsne}}{\partial y_j} + \alpha(t)(y_j^{(t-2)} - y_j^{(t-1)})$$

, η being the learning rate and $\alpha(t)$ the momentum. The second term of $\frac{\mathrm{d}L_{total}}{\mathrm{d}y_j}$ represents an additional force implemented in our aligning version. This formula suggests that λ_i should ideally be scaled inversely with the number of cells in cluster i. Furthermore, the relative magnitude of the first to the second term determines whether the standard $t-$SNE embedding is given a higher weight or the aligning force. λ_i and N_i are cluster specific, but we can redefine $2\frac{\lambda_i}{N_i} = \epsilon$ as a constant value across a query dataset. As such the new update rule becomes:

$$y_j^{(t)} = y_j^{(t-1)} + \eta\frac{\partial L_{tsne}}{\partial y_j} + \alpha(t)(y_j^{(t-2)} - y_j^{(t-1)}) + \epsilon(Y_{r,center_i} - Y_{s,center_i})$$

Computational libraries for efficiently performing t-SNE already exist, so we take advantage of the Python library openTSNE. In this implementation, $\eta = n_{samples}/4$ and $\alpha(t) = 0.5$ for the first 250 iterations, and $\eta = n_{samples}$ and $\alpha(t) = 0.8$ for the remaining iterations. We update the cell positions for each piece of the loss function independently, first performing one embedding iteration with openTSNE, then adjusting cells based on cell type, moving cells towards the corresponding cell type of the embedded reference. In our examples, embedding space spans roughly from -50 to 50 on both the x- and y-axis, and embedding is performed over 750 iterations. We find that an ϵ around 10^{-2} is sufficient for a soft alignment in this case, however some fine-tuning around that range may be necessary. In figure S7, we show, for both examples in the main text, the alignment and preservation scores for several values of ϵ. We see there is a trade-off between alignment improvement and preservation improvement.

2 Supplementary Figures

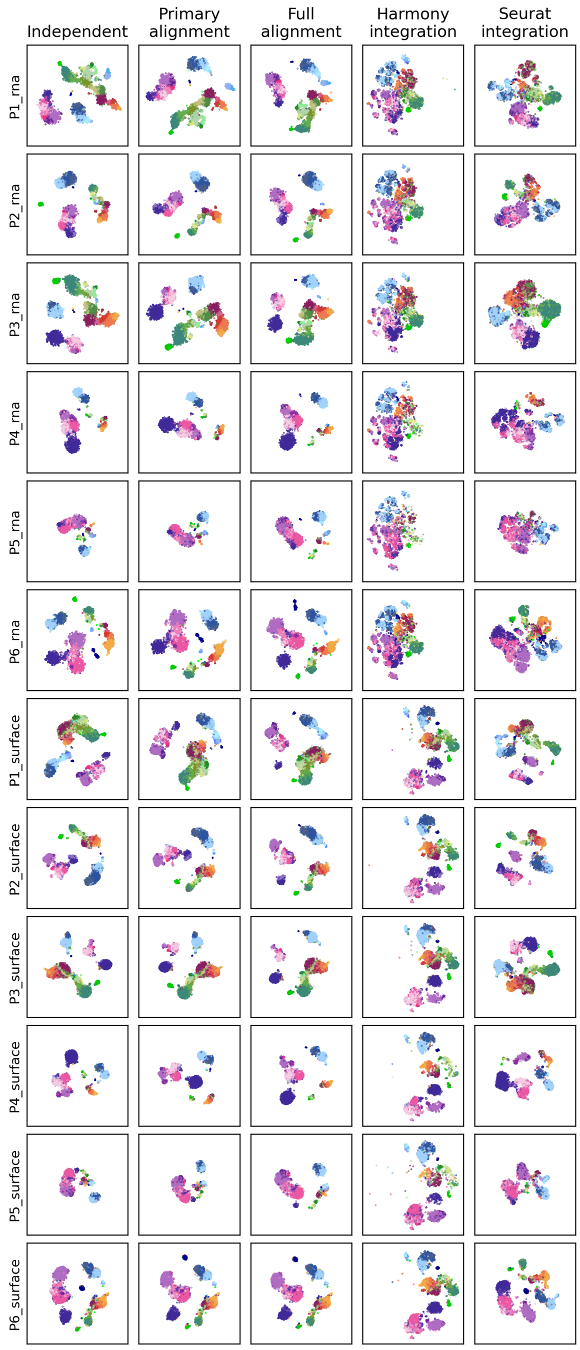

Figure S1: Alignment of scRNA and surface markers for all bone marrow patients

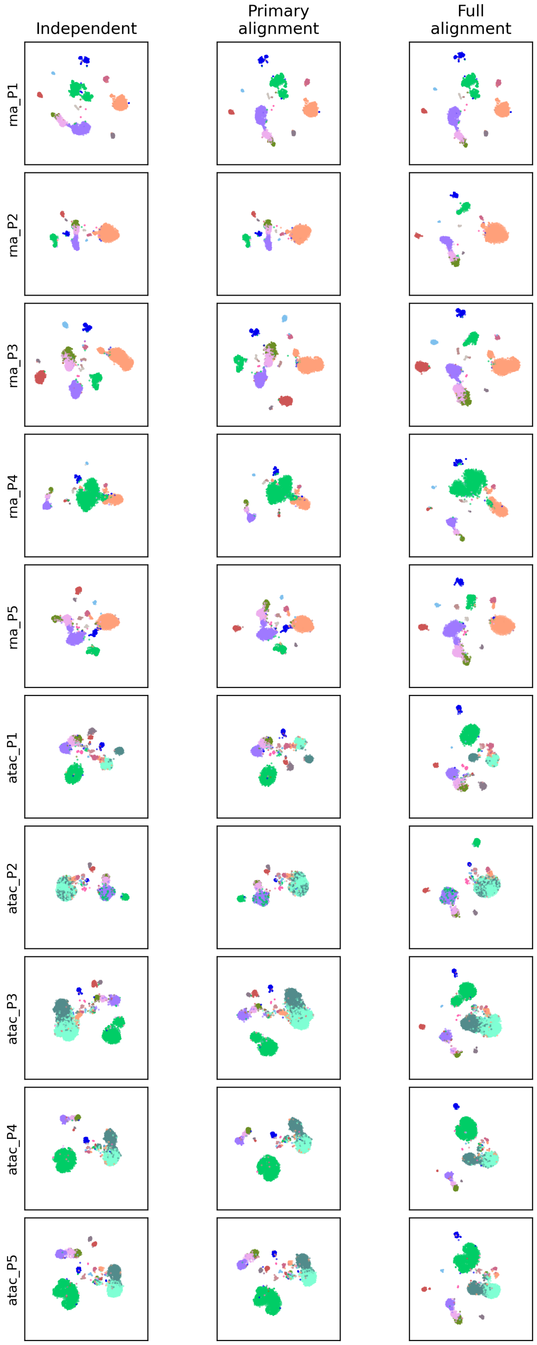

Figure S2: Alignment of scRNA and scATAC markers for all kidney samples.

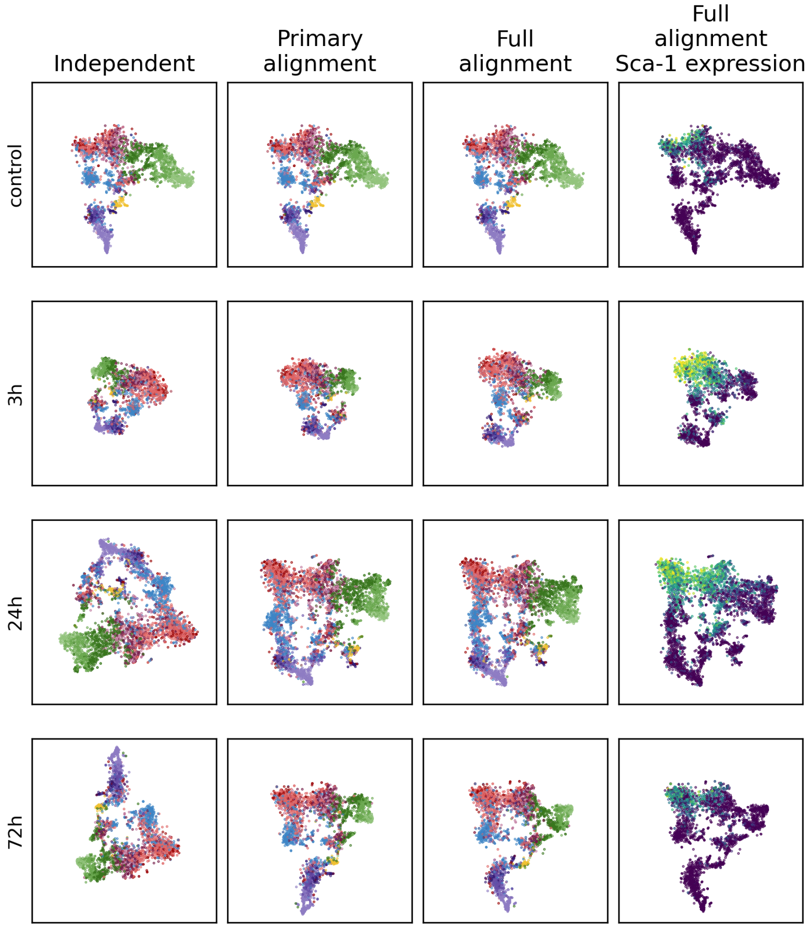

Figure S3: Alignment of hematopoietic cells for several time-points following stimulation by the inflamtory factor IFN-α. We can see that the data manifold at three hours post stimulation is the most different from other time points. We also see that the myeloid branch (green) does not fully recover 72 hours post stimulation. On the right column we highlight the expression of the gene Sca-1, to show how Compound-SNE can be used to visualize differences in gene expression across samples. Cell-type colors are shown in Figure S8.

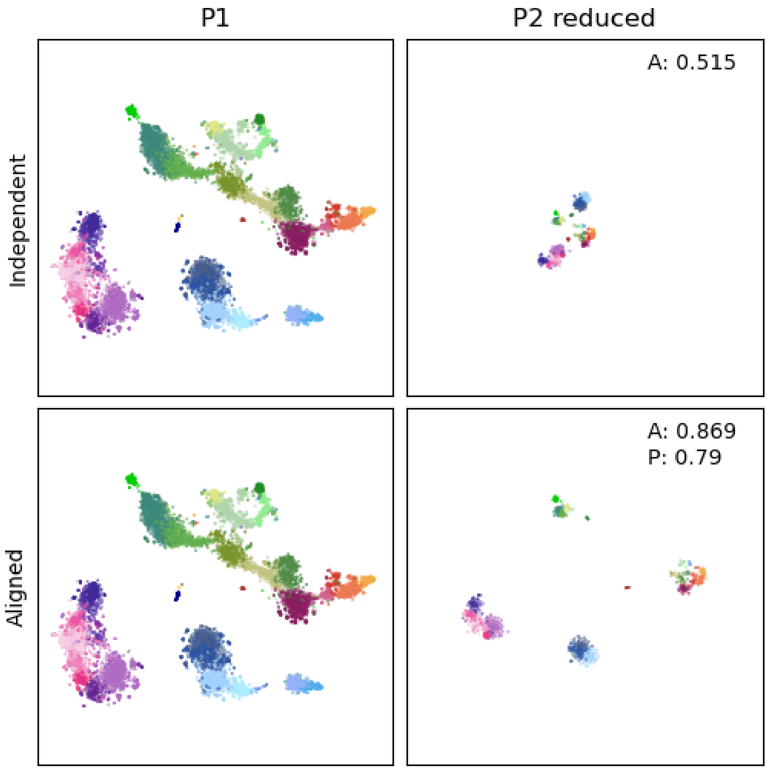

Figure S4: Alignment of scRNA for B1 and B2, with B2 randomly subsampled to 1/10 the size. All embeddings are shown on the same spatial scale.

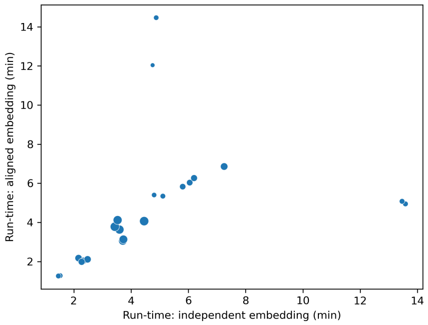

Figure S5: Runtime for independent and aligned embeddings. Each dot represents one sample. Dot size corresponds to sample size. The discrepancy seen between sample size and runtime is due to automatic optimization procedures within openTSNE, which tries to select an appropriate algorithm based on sample size and computational overhead.

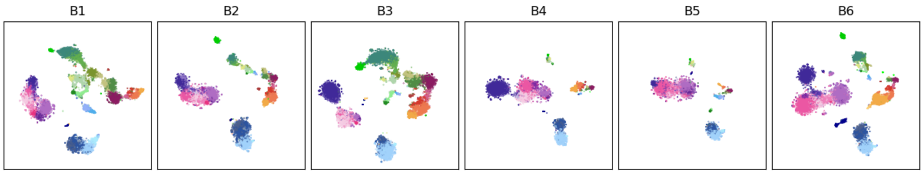

Figure S6: Alignment of bone-marrow scRNA samples via kmeans clustering and MNN.

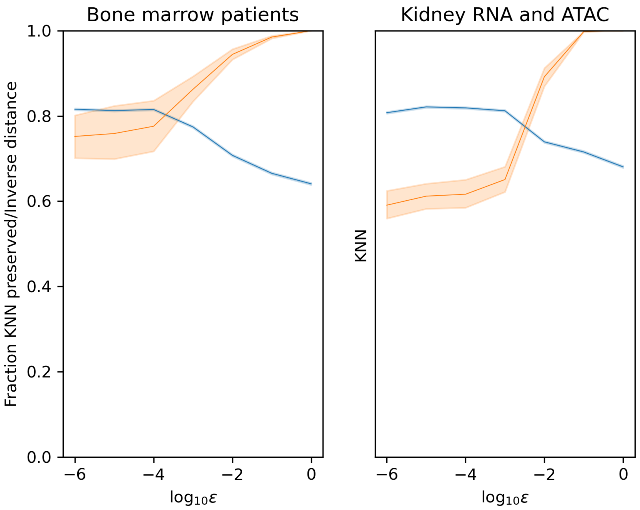

Figure S7: Alignment scores (orange) and preservation scores (blue) for several values of ϵ, with confidence intervals for the 6 bone marrow samples and 5 kidney samples.

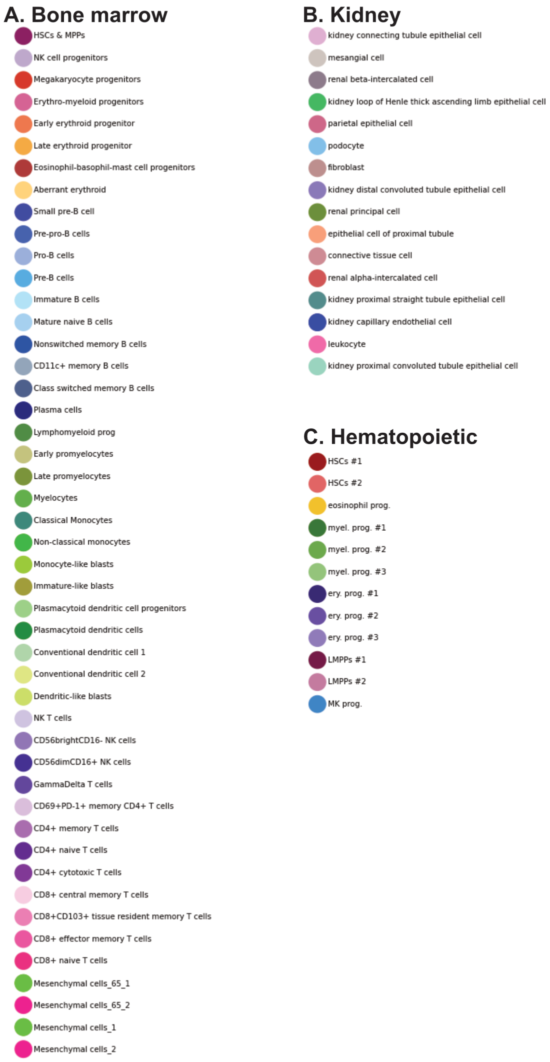

Figure S8: Cell-type legends for the three example datasets: (A) Bone marrow, (B) Kidney, (C) Hematopoietic.
